# Time-resolved correlation of distributed brain activity tracks E-I balance and accounts for diverse scale-free phenomena

**DOI:** 10.1016/j.celrep.2024.114950

**Published:** 2024-10-23

**Authors:** Aditya Nanda, Graham W. Johnson, Yu Mu, Misha B. Ahrens, Catie Chang, Dario J. Englot, Michael Breakspear, Mikail Rubinov

As this paper was originally published on March 24, 2023, a sentence in the experimental model and subject details subsection ‘‘data processing’’ contained an error in the further subsection ‘‘electrophysiology.’’ The text ‘‘All recordings were highpass filtered with a cutoff of 0.5Hz’’ should have instead read ‘‘Macaque anesthesia electrocorticography recordings were highpass filtered with a cutoff of 5Hz, for consistency with the workflow in Solovey et al.^29^ All other recordings were highpass filtered with a cutoff of 0.5Hz.’’ Although the article is too old to update directly with the correct text, we can provide this correction notice to let readers know about the error and give them the updated, correct information.

The authors apologize for the error.

